# Mitophagy in pancreatic cancer: mechanistic insights and implications for novel therapeutic strategies

**DOI:** 10.1038/s41420-026-02948-9

**Published:** 2026-02-05

**Authors:** Zhefang Wang, Zicheng Lyu, Raphael Palmen, Qi Bao, Felix Popp, Qiongzhu Dong, Christiane J. Bruns, Yue Zhao

**Affiliations:** 1 Department of Plastic and Reconstructive Surgery, Second Affiliated Hospital of Zhejiang University School of Medicine, Hangzhou, Zhejiang People’s Republic of China; 2 Department of General, Visceral, Thoracic, and Transplantation Surgery, University Hospital Cologne, Cologne, Germany; 3 Key Laboratory of Whole-Period Monitoring and Precise Intervention of Digestive Cancer, Shanghai Municipal Health Commission, Minhang Hospital, Fudan University, Shanghai, People’s Republic of China

**Keywords:** Pancreatic cancer, Cancer therapeutic resistance

## Abstract

Pancreatic ductal adenocarcinoma (PDAC) presents significant treatment challenges, primarily due to its propensity for developing resistance to therapeutic interventions. While the underlying mechanisms remain elusive, they are closely associated with mitochondrial adaptation in response to treatment. Mitophagy, a selective subtype of autophagy that eliminates damaged or surplus mitochondria, is crucial for tumorigenesis, progression, and treatment resistance in cancers. This review discusses the intricate regulatory pathways of mitophagy in PDAC, focusing on the PINK1/Parkin pathway and receptor-mediated pathways. Furthermore, it explores the therapeutic potential of targeting mitophagy to increase the effectiveness of existing treatments and improve patient survival. Current evidence indicates that combining mitophagy inhibition with conventional chemotherapy yields promising yet inconsistent results, which may be attributed to the context-dependent functions of mitophagy and a lack of specific inhibitors. This review highlights the therapeutic potential of targeting mitophagy in PDAC and underscores the necessity for biomarker-driven patient stratification and the development of pathway-specific modulators in future clinical efforts.

## Facts

- The PINK1/Parkin pathway and receptor-mediated pathways regulate mitophagy through distinct mechanisms.
- Mitophagy plays context-dependent and pathway-specific roles in PDAC.
- Mitophagy inhibition shows promising yet inconsistent results in clinical trials, highlighting the need for biomarker-driven patient stratification.

## Open questions

- How do the PINK1/Parkin and receptor-mediated pathways interact and compensate for each other in the specific context of PDAC treatment resistance?
- Can we develop direct, specific, and potent small-molecule inhibitors or inducers of distinct mitophagy pathways?
- What are the most reliable biomarkers to stratify PDAC patients for mitophagy-targeted combination therapies?

## Introduction

Pancreatic ductal adenocarcinoma (PDAC) is one of the most lethal malignancies in the world and ranks as the sixth cause of cancer death, accounting for 5% of all cancer deaths worldwide [1, 2]. The 5-year survival rate of PDAC is less than 10%, while the mortality rate continues to gradually rise [1]. To date, chemotherapy remains the primary approach for managing PDAC, aside from curative surgery. Recently, targeted therapy aimed at the KRAS-G12D mutation has demonstrated promising results in preclinical studies [3]. However, acquired resistance to treatment—whether chemotherapy or targeted therapy—remains the most significant challenge in the management of PDAC [4, 5]. While the underlying mechanisms remain elusive, they are closely associated with mitochondrial adaptation in response to treatments [6–8]. Mitochondria are versatile organelles that not only generate the majority of a cell’s energy but also play critical roles in metabolic regulation, calcium signaling, redox homeostasis, and apoptosis [9]. In cancer cells, mitochondria are often impaired, which prompts reliance on glycolysis for energy even in aerobic conditions-a phenomenon known as the “Warburg effect” [10]. Recent research suggests that mitochondria contribute significantly to the treatment resistance of PDAC by influencing apoptosis, metabolism, mitochondrial DNA (mtDNA) processes, and mitochondrial dynamics [11, 12]. Mitophagy, a selective type of autophagy, is essential for removing damaged or surplus mitochondria, and is thus crucial for maintaining mitochondrial homeostasis [13]. PDAC typically exhibits increased mitochondrial fragmentation and elevated mitochondrial fission compared to normal tissues, indicating a heightened demand for mitochondrial quality control via mitophagy [14, 15]. Generally, cancer cells use mitophagy to rapidly eliminate damaged mitochondria, thereby promoting tumor progression and mediating drug resistance in various cancers, including PDAC [16]. The impact of targeting mitophagy on treatment resistance in PDAC is an area of interest. Combined therapeutic strategies that target mitophagy may enhance the efficacy of chemotherapeutic agents like gemcitabine and paclitaxel, potentially extending the survival of PDAC patients [17, 18].

In this review, we first delineate the core mechanisms of mitophagy, then explore its complex, context-dependent roles in PDAC pathogenesis and treatment resistance, and finally critically evaluate the emerging therapeutic strategies of targeting mitophagy, concluding with future perspectives and challenges.

## Mitophagy pathways and mechanisms

**Fig. 1:**
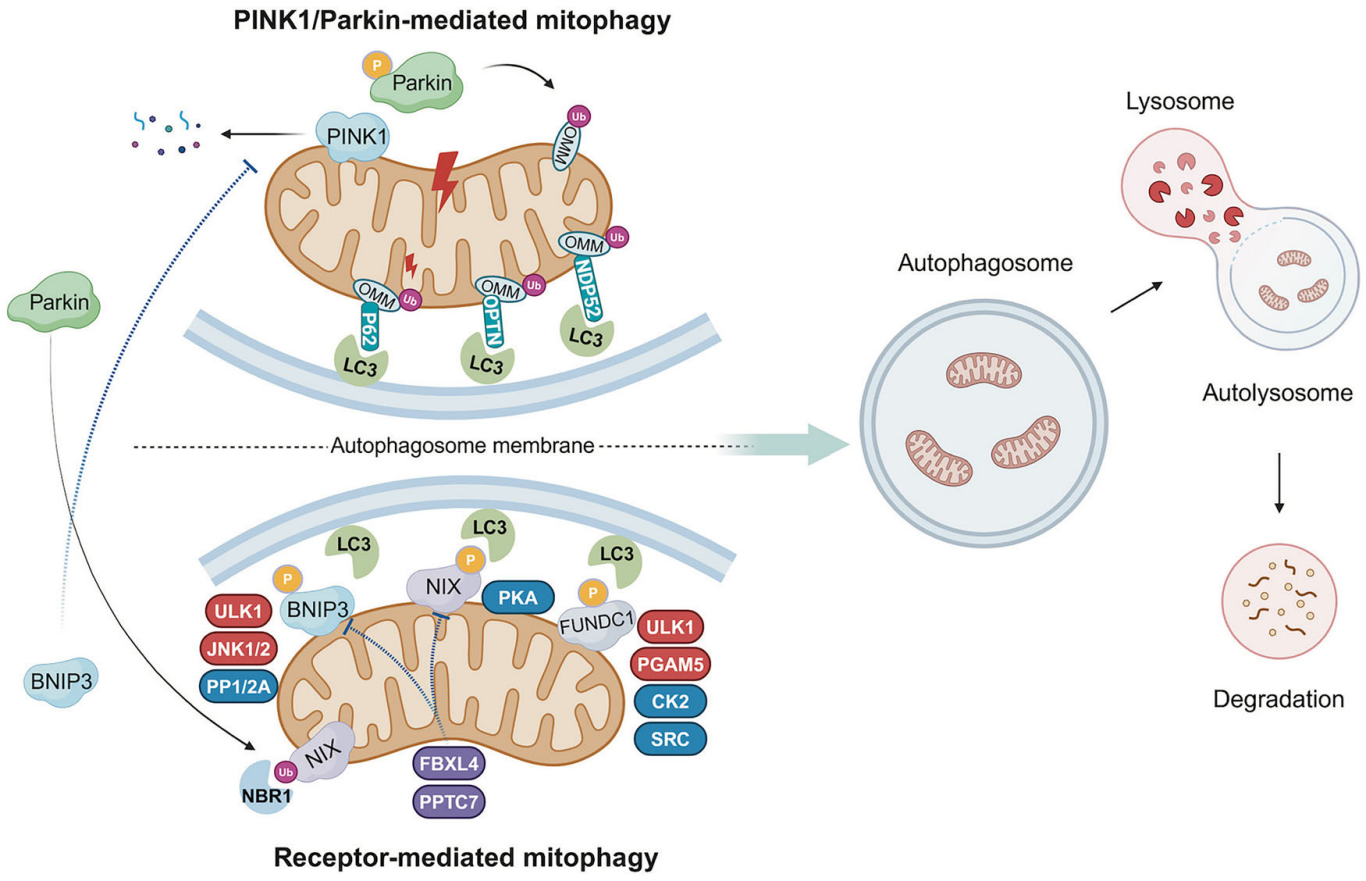
Schematic illustration of PINK1/Parkin-mediated and receptor-mediated mitophagy. Upon mitochondrial depolarization, PINK1 stabilizes on the outer mitochondrial membrane (OMM) and recruits and phosphorylates Parkin. This leads to the ubiquitination of OMM proteins, including MFN1/2, MIRO1, and VDAC1. Ubiquitinated OMM proteins then interact with cargo receptors such as P62/SQSTM1, Optineurin (OPTN), and NDP52, which facilitate the formation of autophagosomes through interactions with LC3. BNIP3, NIX and FUNDC1 are the most studied mitochondrial cargo receptors (MCR), which facilitate the targeting of mitochondria to autophagosomes through direct interaction with LC3. These MCRs are regulated post-translationally by kinases and phosphatases, such as ULK1, PKA, CK2, PGAM5 and PPTC7. Crosstalk occurs between the two pathways; for example, Parkin can ubiquitinate MCRs like NIX, and BNIP3 can stabilize PINK1. Ultimately, tagged mitochondria are engulfed by autophagosomes, which fuse with lysosomes to form autolysosomes for degradation.

Autophagy, a highly conserved catabolic mechanism, involves the delivery of cellular components to lysosomes for degradation and recycling, providing essential energy and macromolecules [19]. This process can be either non-selective or selective, with mitophagy being one of the most characterized type of the latter [20]. Mitophagy (termed mitochondrial autophagy) is a selective autophagic process that targets dysfunctional or redundant mitochondria through stress-responsive mechanisms. This evolutionarily conserved pathway functions as an adaptive survival response to cellular stressors such as oxidative damage, hypoxia, and nutrient deprivation, while serving as a critical mitochondrial quality control mechanism. By eliminating compromised mitochondria, mitophagy preserves cellular homeostasis and mitigates reactive oxygen species (ROS) overproduction [21]. The classical regulatory mechanism of mitophagy is mediated through PINK1/Parkin-dependent ubiquitination in mitochondria [22]. Additionally, increasing attention is being given to ubiquitin-independent pathways of mitophagy, which involve a growing number of mitochondrial cargo receptors (MCRs), such as BNIP3, BNIP3L/NIX, and FUNDC1 [21]. Under stress conditions, these receptors localize to the mitochondria and promote mitophagy through direct interaction with processed microtubule-associated protein 1 light chain 3 (LC3) via their conserved LC3 interaction regions (LIR) domain. This interaction facilitates the encapsulation of targeted mitochondria within autophagosomes, which subsequently fuse with lysosomes to form autolysosomes, thereby enabling the degradation of the mitochondrial cargo [23, 24] (Fig. 1).

### PINK1/Parkin-mediated mitophagy

The PINK1/Parkin pathway is the most extensively characterized mechanism regulating mitophagy in mammals [25]. PINK1 is a serine/threonine protein kinase that acts as a sensor of mitochondrial damage, whereas Parkin is an E3 ubiquitin ligase that functions as a signal amplifier. Under healthy conditions, PINK1 is translocated to the inner mitochondrial membrane (IMM), where it is cleaved by mitochondrial processing peptidase (MPP) and the presenilin-associated rhomboid-like (PARL) protease, and subsequently degraded through the N-end rule pathway [26]. When mitochondria are depolarized, the loss of mitochondrial membrane potential stabilizes PINK1 on the outer mitochondrial membrane (OMM), where it recruits and phosphorylates Parkin at serine 65 within its ubiquitin-like (UBL) domain, amplifying Parkin’s recruitment to the OMM [25]. This activation leads to the ubiquitination and phosphorylation of OMM proteins, such as MFN1/2, MIRO1, and VDAC1 [27]. These proteins then interact with autophagic cargo receptors such as P62/SQSTM1, Optineurin (OPTN), and NDP52, which serve as molecular adaptors that link ubiquitinated mitochondria to autophagosomes through interaction with LC3 via their LIR motifs [27, 28]. PINK1/Parkin-mediated mitophagy is the major mechanism for eliminating depolarized mitochondria. In addition, PINK1 could also mediate mitophagy through interacting with other mitochondrial E3 ubiquitin ligases, including ARIH1 [29], SIAH1 [30], and MUL1 [31]. Furthermore, PINK1/Parkin-dependent mitophagy can also be activated by the accumulation of unfolded proteins within the mitochondrial matrix, highlighting a novel trigger beyond mitochondrial depolarization [32].

### Receptor-mediated mitophagy

BNIP3 and BNIP3L (also known as NIX) are OMM proteins that belong to the BH3-only Bcl-2 protein family. They are both structurally and functionally homologous, sharing similar roles in regulating apoptosis and mitophagy [33, 34]. Distinct from PINK1/Parkin-mediated mitophagy, the processes driven by BNIP3 and NIX do not rely on mitochondrial ubiquitination. BNIP3 and NIX facilitate the targeting of mitochondria to autophagosomes through direct interactions with LC3, which is located on the cytosolic surface of autophagosomal membranes, utilizing both typical and atypical LIR motifs [35]. Homo-dimerization of BNIP3 or NIX is required for efficient LC3 interactions [36]. The molecular mechanisms underlying BNIP3/NIX-mediated mitophagy are not completely understood, but hypoxia is recognized as a typical stressor that induces their activation. Both BNIP3 and NIX are transcriptionally regulated by HIF-1α [37]. Additionally, BNIP3 and NIX are established transcriptional targets of p53, with NIX being upregulated [38] and BNIP3 being repressed [39] by this tumor suppressor under hypoxic conditions via direct DNA binding. Compromised p53 function, such as through mutation, abrogates its regulatory control over BNIP3 and NIX. Furthermore, BNIP3 and NIX are also post-translationally regulated by various kinases and phosphatases, including ULK1 [40], JNK1/2 [41], PP1/2A [41], and PKA [42], which modulate their protein stability and interactions with LC3. For BNIP3, the common phosphorylation sites are Ser17 and Ser60/Thr66 [40, 41], while for NIX, phosphorylation typically occurs at Ser34/35 and Ser212 [42, 43]. Recently, a new mechanism regulated by SCF^FBXL4^-mediated ubiquitination of BNIP3 and NIX was reported [44]. This regulation requires PPTC7, a conserved mitochondrial matrix PP2C phosphatase, which acts as a scaffold for assembling the substrate-PPTC7-SCF^FBXL4^ holocomplex that then facilitates degradation of BNIP3 and NIX [45, 46].

FUNDC1 is a ubiquitously expressed OMM protein. Similar to BNIP3 and NIX, FUNDC1 promotes hypoxia-induced mitophagy. However, the regulation of FUNDC1 by hypoxia predominantly occurs through post-translational modifications, mainly phosphorylation and dephosphorylation, which are crucial for its interaction with LC3 and the subsequent regulation of mitophagy [47–49]. In general, the phosphorylation of Ser13 and Tyr18 on FUNDC1 by CK2 and SRC kinases, respectively, weakens the LC3-FUNDC1 interaction [47, 48]. Conversely, PGAM5 catalyzes the dephosphorylation of Ser13 on FUNDC1 and restores LC3-FUNDC1 interaction [49]. Meanwhile, ULK1-mediated phosphorylation of Ser17 on FUNDC1 facilitates its interaction with LC3 and enhances mitophagy [50].

### Pathway crosstalk and contextual redundancy

Mitophagy pathways exhibit redundancy and may interact with each other or be distinctly necessary in response to specific stress. Generally, PINK1/Parkin-mediated mitophagy is triggered by mitochondrial depolarization, whereas mitophagy mediated by BNIP3, NIX, and FUNDC1 is primarily activated by conditions such as hypoxia [51]. This delineates a preference of the PINK1/Parkin pathway for targeting and eliminating dysfunctional mitochondria, whereas the receptor pathway primarily addresses excess mitochondria [52, 53]. Recently, NIX has also been implicated in PINK1/Parkin-mediated mitophagy, acting as a substrate of Parkin. The PARK2 (Parkin)-mediated ubiquitination of NIX triggers the recruitment of the selective autophagy receptor NBR1 to the mitochondrial surface, thereby facilitating degradation of mitochondria [54]. Additionally, BNIP3 has been shown to facilitate the accumulation of full-length PINK1 on OMM by inhibiting PINK1 kinase’s proteolytic cleavage, thus enhancing PINK1/Parkin-mediated mitophagy [55]. Under varying conditions, these pathways tend to operate synergistically rather than in isolation, each exhibiting distinct roles and preferences based on the cellular context and the specific nature of mitochondrial dysfunction or abundance [56]. The redundancy and complex interplay among various mitophagy pathways highlight the intricacy of mitochondrial adaptation to stress, underscoring the need for further investigation into these interactions.

## Mitophagy in PDAC

**Fig. 2:**
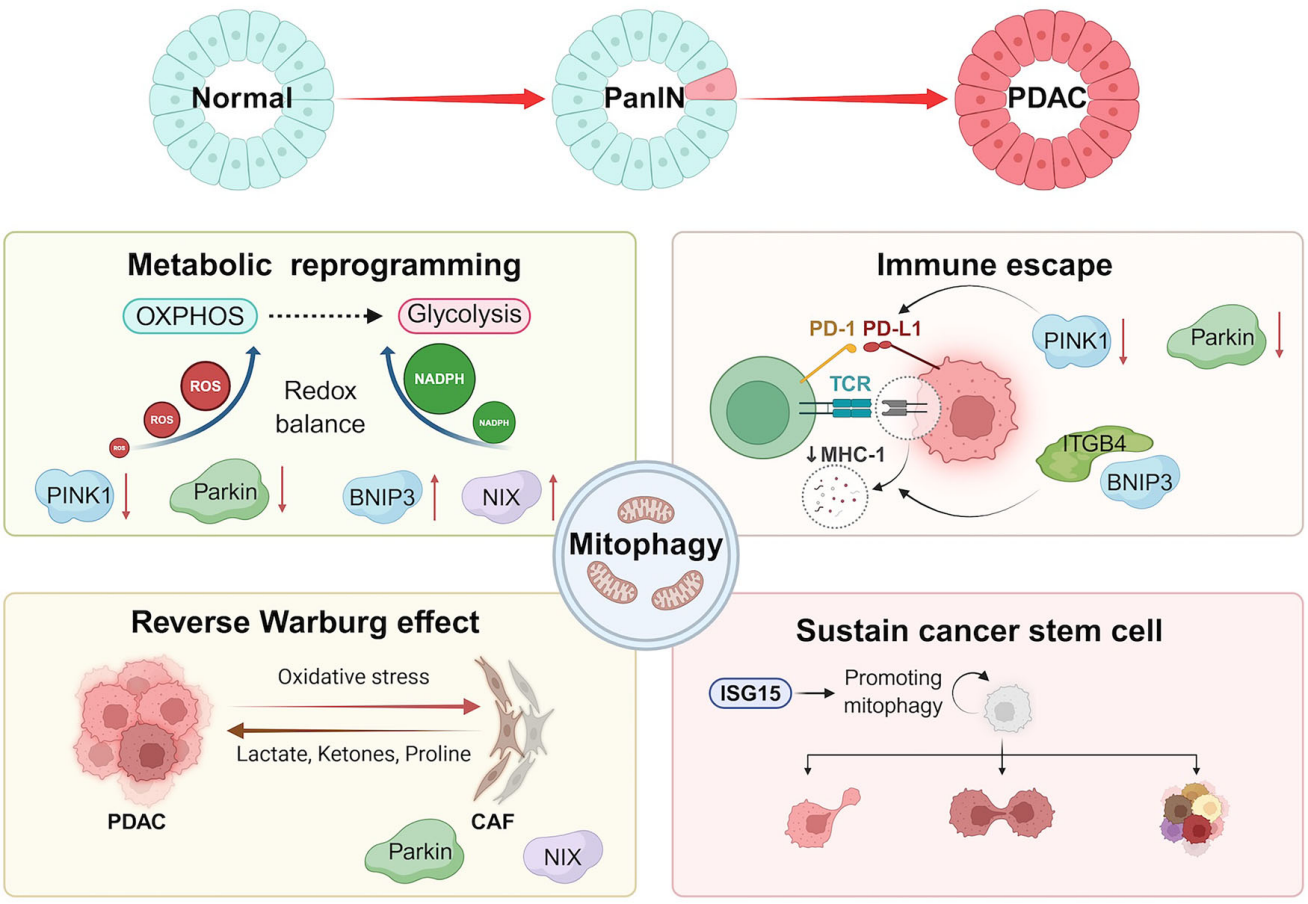
The role of mitophagy in the pathogenesis of PDAC. (Upper left) During pancreatic intraepithelial neoplasia (PanIN) progression, PINK1/Parkin-mediated mitophagy is suppressed, while receptor-mediated mitophagy (e.g., via NIX) is upregulated. This promotes tumor initiation and progression by enhancing redox homeostasis and driving a metabolic shift from oxidative phosphorylation (OXPHOS) to glycolysis. (Lower left) Cancer-associated fibroblasts (CAFs) undergo mitophagy and autophagy via the reverse Warburg effect. Cancer cell-induced oxidative stress triggers metabolic reprogramming in CAFs toward aerobic glycolysis, leading to the secretion of metabolites (e.g., lactate, ketones, proline) that support tumor growth. (Upper right) PINK1/PARK2 deletion upregulates PD-L1, and the ITGB4/BNIP3 complex promotes autophagic degradation of MHC-I, facilitating immune escape. (Lower right) ISG15-mediated ISGylation supports mitophagy in pancreatic cancer stem cells (CSCs), maintaining their metabolic plasticity and self-renewal capacity.

PDAC cells display prominent mitochondrial alterations, including elevated mtDNA mutation rates, enhanced mitochondrial fragmentation, and increased fission activity compared to normal pancreatic tissues [14, 15]. The clearance of damaged mitochondria is critical for preserving mitochondrial homeostasis. This process, primarily mediated by mitophagy, not only supports metabolic plasticity and reprogramming in PDAC cells but also fosters tumorigenesis and confers resistance to apoptosis [57]. The multifaceted roles of mitophagy in PDAC development and progression are summarized in Fig. 2.

### Dual faces of mitophagy in tumor initiation and progression

Oncogenic KRAS mutation plays critical roles in the initiation and maintenance of PDAC progression and is associated with alterations in mitochondrial morphology and function. However, the role of mitophagy regulators such as PINK1/PRKN, BNIP3, and NIX in pancreatic tumorigenesis and progression is dynamic and intricate, as demonstrated in transgenic PDAC mouse models [53, 58]. Li et al. demonstrate that PINK1 and PRKN deficiency accelerates pancreatic tumorigenesis through mitochondrial iron accumulation, which accelerates KRAS-driven PDAC initiation by enhanced ROS formation, immune suppression and metabolic reprogramming from OXPHOS to glycolysis [58]. The tumor suppressor functions of PINK1 and PRKN are primarily due to selective clearance of depolarized mitochondria through mitophagy, thereby preventing Warburg metabolism and excess ROS, which is essential for tumorigenesis [58, 59].

Conversely, Humpton et al. demonstrate that NIX-mediated mitophagy functions as a novel effector pathway of oncogenic KRAS, promotes the elimination of functional mitochondrial content to stimulate cell proliferation and augments redox homeostasis by increasing glycolytic metabolism and augmenting redox robustness [53]. Deletion of NIX results in an increased mitochondrial load in PanIN, thereby delaying tumor progression from PanIN to PDAC. This may be rationalized by the hypoxic and nutrient-poor tumor microenvironment in PDAC, which is characterized by dense desmoplasia. Notably, BNIP3 was upregulated in NIX knockout mice, suggesting a compensatory mechanism for receptor-mediated mitophagy under hypoxia. However, BNIP3’s expression pattern diverges from that of NIX during PDAC progression; it is induced in early pre-malignant stages but is downregulated in advanced tumors [60]. Hypermethylation of the BNIP3 promoter was observed in BNIP3-negative PDAC cell lines, which is thought to be associated with abnormal methylation mediated by DNMT3β and DNMT1 [61]. Restoration of BNIP3 inhibits the proliferation of PDAC cell lines, while the underlying mechanism is still unknown.

These findings suggest that PINK1/Parkin-mediated and receptor-mediated mitophagy play distinct roles in PDAC tumorigenesis and progression, potentially by selectively eliminating damaged versus functional mitochondria to meet stage-specific ROS requirements, thereby promoting a metabolic shift from OXPHOS to glycolysis. Further investigation is necessary to fully elucidate these pathway-specific mechanisms.

### Mitophagy in cancer stem cells

In addition, mitophagy plays a critical role in sustaining the cancer stem cell (CSC) phenotype, which is essential for self-renewal and chemotherapy resistance in cancers, including PDAC [62]. The ubiquitin-like modifier interferon-stimulated gene 15 (ISG15) and the process of ISGylation are crucial for maintaining the metabolic plasticity of pancreatic CSCs by promoting mitophagy [63]. PDAC patients with high ISG15 levels were enriched with CSC-related pathways, including epithelial-mesenchymal transition (EMT) and OXPHOS [63]. This further highlights the therapeutic potential of targeting mitophagy to eradicate CSCs and overcome treatment resistance in PDAC.

### Mitophagy in tumor microenvironment interactions

The tumor microenvironment (TME), a dynamic ecosystem comprising extracellular matrix (ECM) components, stromal cells and immune infiltrates, is known to influence tumor development, progression, metastasis and therapy resistance [64]. Among its cellular constituents, cancer-associated fibroblasts (CAFs) have emerged as pivotal regulators of TME plasticity. One tumor-enhancing characteristic found in CAFs is the reverse Warburg effect [65, 66]. Specifically, cancer cells induce oxidative stress in neighboring CAFs, triggering autophagy and mitophagy, which in turn reinforce the CAFs to undergo metabolic reprogramming towards aerobic glycolysis. As a result, the CAFs supply high-energy metabolites such as lactate and ketones to tumor cells, promoting cancer growth and progression [67]. In PDAC, a deficiency in autophagy has been associated with an increased activation of CAFs, primarily through reduced proline biosynthesis and collagen production [68]. It was shown that autophagy supports proline synthesis by regulating NAKD2 in a mitophagy-dependent manner. Inhibition of mitophagy in the stromal compartment, for example, by targeting PRKN, led to a decrease in tumor weight.

Mitophagy also plays a critical role in shaping immune responses within the TME. The deletion of PINK1/PARK2 leads to upregulation of PD-L1 during PDAC tumorigenesis [58]. Recently, Zhou et al. reported that activating ITGB4/BNIP3 could promote the phagocytosis of MHC-I by autophagosomes, thus promoting immune escape in PDAC [69]. In addition, MHC-I molecules could be selectively trapped and degraded in lysosomes through an autophagy-dependent mechanism involving the autophagy cargo receptor NBR1 [70]. Interestingly, mitochondria carrying mtDNA mutations can transfer from cancer cells to tumor-infiltrating lymphocytes (TILs), and these transferred mitochondria fail to undergo mitophagy. As a result, TILs acquiring cancer-derived mtDNA develop metabolic abnormalities and senescence with impaired effector and memory functions [71]. Though this has been demonstrated only in melanoma and non–small cell lung cancer, the direct regulation of mitophagy in immune cells within the PDAC tumor microenvironment remains largely unexplored. Nevertheless, a bioinformatic analysis revealed the correlation of PINK1 expression with immune cell infiltration in cancers [72]. Together, these findings highlight mitophagy as a therapeutic target within the tumor microenvironment.

## Mitophagy as an engine of therapy resistance and a therapeutic target

### Therapy resistance

**Fig. 3:**
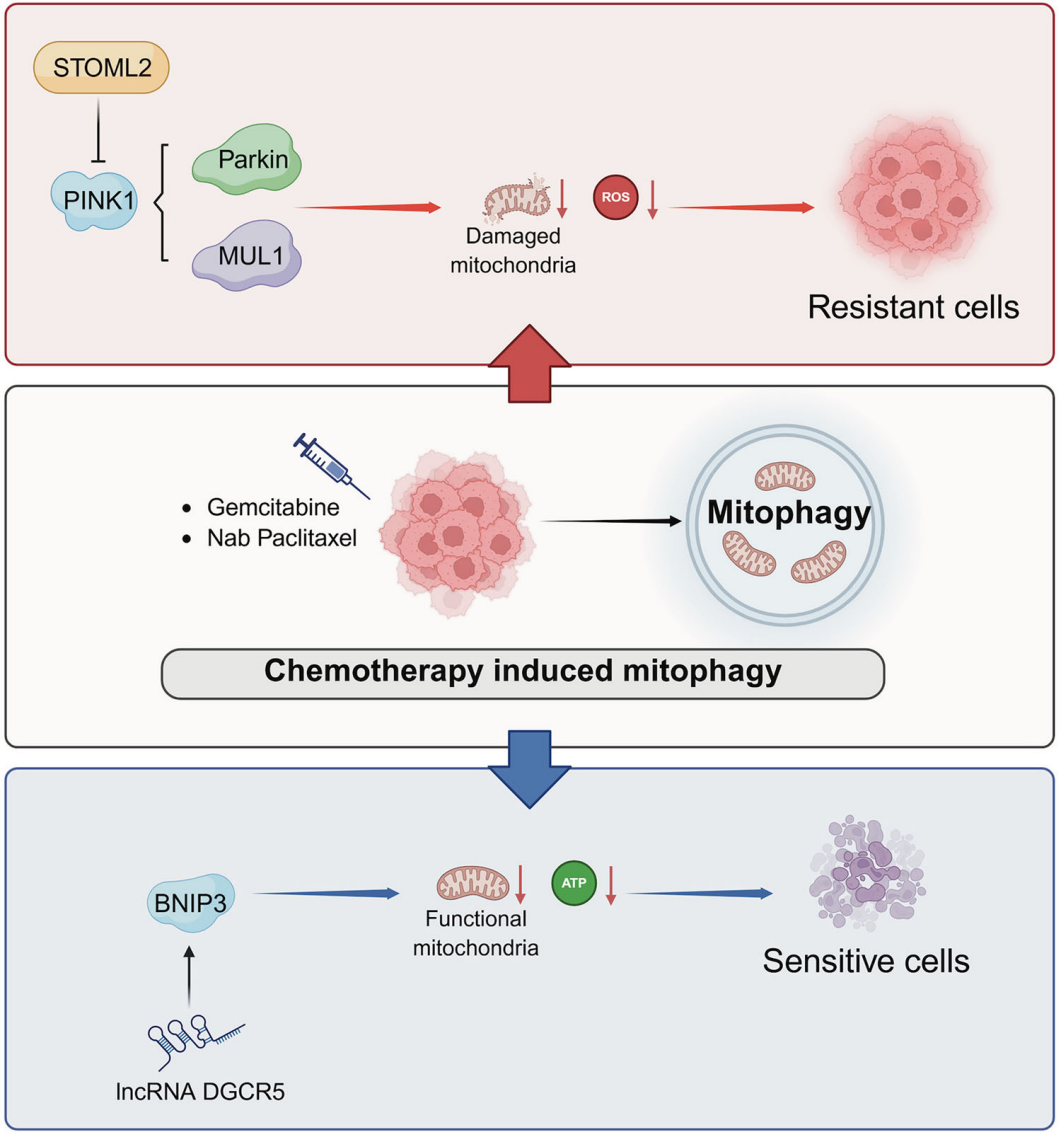
Dual roles of mitophagy in PDAC therapy resistance. PINK1-mediated mitophagy (both Parkin-dependent and independent) promotes resistance to chemotherapy-induced cell death. In contrast, BNIP3 upregulation (e.g., via lncRNA DGCR5) enhances apoptosis and chemosensitivity. These opposing effects underscore the context-dependent nature of mitophagy in PDAC treatment resistance, necessitating further investigation into pathway-specific regulatory mechanisms.

It has long been recognized that mitochondrial quality control and turnover play important roles in the treatment resistance of PDAC. Mitophagy generally facilitates the rapid clearance of damaged mitochondria induced by cytotoxic agents, thereby preventing the accumulation of dysfunctional mitochondria, mitigating oxidative stress, and inhibiting the release of pro-apoptotic factors. Several studies have shown that gemcitabine treatment induces active PINK1-mediated mitophagy and contributes to the chemoresistance of PDAC [73–75]. For instance, one study demonstrates that STOML2, a mitochondrial inner membrane protein critical for mitochondrial stability, inhibits mitophagy by degrading PINK1 through PARL stabilization in PDAC cells [73]. This action prevents gemcitabine-induced PINK1-dependent mitophagy and increases chemosensitivity in PDAC cells. Additionally, PINK1/Parkin-mediated mitophagy is also observed following treatment with Rocaglamide A, a compound extracted from the plant *Aglaia elliptifolia*, serving as a bypass mechanism to limit Rocaglamide A-induced apoptosis [76]. Meanwhile, gemcitabine-induced mitophagy may occur independently of Parkin and instead be mediated by alternative mitochondrial E3 ligases, such as MUL1 [75]. In summary, PINK1-mediated mitophagy, whether Parkin-dependent or independent, may confer resistance to chemotherapy-induced cell death in PDAC cells (Fig. 3).

Conversely, there is also a perspective that promoting mitophagy could enhance chemotherapy-induced cell death under specific circumstances. This view suggests that excessive mitophagy might lead to loss of functional mitochondria, thereby exacerbating energy deficits and triggering apoptotic pathways. Supporting the potential benefits of mitophagy in chemosensitivity, decreased expression of BNIP3 is associated with intrinsic chemoresistance to gemcitabine in PDAC cell lines [60, 77]. Elevated expression of BNIP3 by lncRNA DGCR5 promotes cell apoptosis in PDAC cells (Fig. 3) [78]. One point supporting BNIP3-mediated mitophagy in chemosensitivity is that NIX expression is widely elevated in PDAC patients [53]. Dual activation of BNIP3 and NIX may lead to excessive mitophagy, given their similar structure and function, as indicated by the compensatory upregulation of BNIP3 when NIX is deleted [53]. Furthermore, double knockout of BNIP3 and NIX completely abolishes mitophagy, whereas single knockout of either BNIP3 or NIX only slightly decreases mitophagy [79]. However, more conclusive evidence is needed to elucidate the regulatory pathway-specific and context-dependent roles of mitophagy in the treatment resistance of PDAC.

### Therapeutic strategies: inhibiting mitophagy

**Fig. 4:**
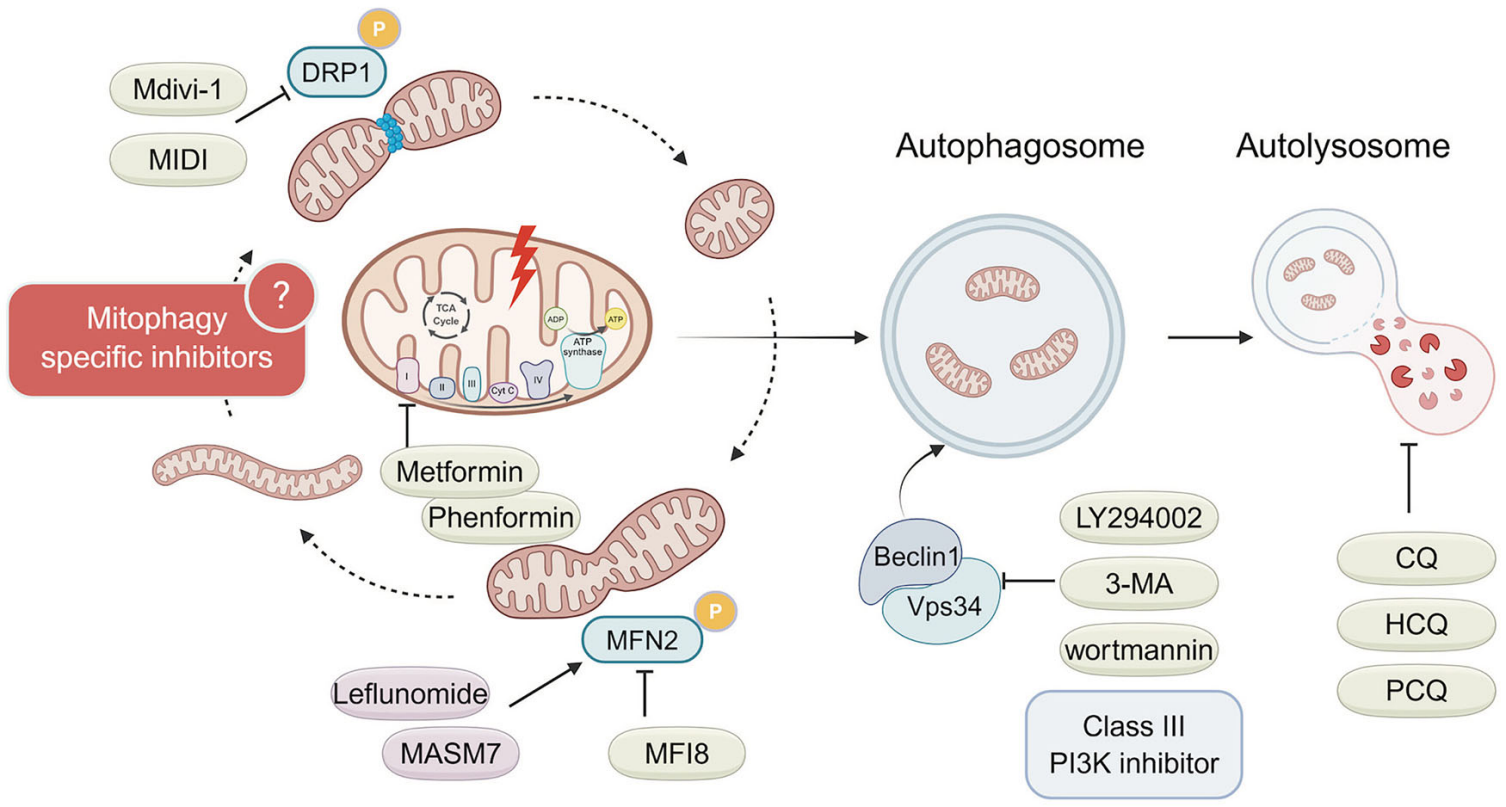
Therapeutic strategies for modulating mitophagy in PDAC. Although direct activators or inhibitors of PINK1/Parkin- or receptor-mediated mitophagy are currently lacking, mitophagy can be modulated indirectly through several strategies. Inhibition of mitophagy: Small molecules targeting mitochondrial dynamics (e.g., DRP1, MFN2), class III PI3K, or autophagosome–lysosome fusion can suppress mitophagic flux. Promotion of mitophagy: Biguanides (e.g., metformin, phenformin) inhibit mitochondrial complex I, inducing mitophagy. These indirect approaches offer potential avenues for therapeutic intervention in mitophagy-associated PDAC treatment resistance.

Combination chemotherapy is crucial for the management of PDAC, yet the objective response rates are still quite low [80]. Current treatments, including chemotherapy and targeted therapy, often encounter resistance over time due to adaptive cellular mechanisms like mitophagy. Targeting mitophagy, either through inhibition or promotion, represents a novel strategy for combination therapy in PDAC patients, potentially augmenting the efficacy of existing chemotherapy regimens. However, compounds that directly activate or inhibit either PINK1/Parkin or receptor-mediated mitophagy are currently lacking [81]. Consequently, mitophagy is primarily regulated indirectly in PDAC by targeting factors such as mitochondrial integrity, mitochondrial dynamics, autophagosome formation, and autolysosome formation [16] (Fig. 4).

#### Targeting mitochondrial dynamics

Mitochondrial morphology is dynamically regulated by the processes of fission and fusion, both of which play critical roles in the regulation of mitophagy [82]. Among the proteins involved in mitochondrial dynamics, dynamin-related protein 1 (DRP1) and mitofusin 2 (MFN2) are the most extensively studied for their key roles in PDAC [83]. DRP1-mediated mitochondrial fission is required for KRAS-driven transformation, tumor growth and metastasis in PDAC [14, 84]. Mdivi-1 is a small molecule initially identified as a selective inhibitor of DRP1, as it inhibits DRP1 GTPase activity and its self-assembly into rings [85]. Inhibition of mitochondrial fission by mdivi-1 impairs mitophagy, resulting in the accumulation of dysfunctional mitochondria. This subsequently impedes tumorigenicity and invasiveness while enhancing chemosensitivity in PDAC, particularly in CD133 + CSCs [76, 86]. However, the specificity of mdivi-1 for DRP1 and its effect on mitochondrial fission have been challenged. Bordt et al. assert that mdivi-1 does not alter mitochondrial morphology in mammalian cells [87]. Conversely, mdivi-1 reversibly inhibits respiration at mitochondrial complex I, which is independent of DRP1 action [87, 88]. Further investigations into the effects of mdivi-1 on DRP1 function and mitophagy in PDAC are required. Recently, new DRP1 GTPase inhibitors, Drpitor1 and Drpitor1a, were identified through in silico screening and may serve as novel candidates for mitophagy inhibition [89]. MFN2, a key mediator of mitochondrial fusion, facilitates mitophagy through the recruitment of Parkin when phosphorylated by PINK1 [90, 91]. In PDAC, MFN2-mediated mitochondrial fusion helps to normalize the fragmented mitochondria by inducing mitophagy, which in turn suppresses tumor growth and improves survival in preclinical models [92]. Additionally, leflunomide, an FDA-approved drug for arthritis, has been shown to inhibit the growth of PDAC tumors by inducing MFN2 expression and subsequent mitophagy [92]. Lidamycin, an antitumor antibiotic, has also been reported to induce mitophagy in PDAC cells by regulating the expression of MFN2 [93]. Knockdown of MFN2 rescues cells from lidamycin-induced apoptosis by attenuating the mitophagy triggered by lidamycin. Unlike DRP1-regulated mitophagy, these results suggest that MFN2-regulated mitophagy exhibits tumor-suppressive effects in PDAC.

Compared to inhibitors identified through functional screening, compounds developed via structure-based design typically exhibit greater potency and specificity for their targets. Recently, a novel and potent mitochondrial fission inhibitor, MIDI, has been identified and may represent a promising candidate for mitophagy inhibition [94]. Additionally, new small-molecule compounds have been developed to modulate MFN1/2 activity: MASM7, an activator, and MFI8, an inhibitor [95]. These MFN1/2 modulators enable the temporal and reversible control of mitochondrial fusion, offering a therapeutic strategy for PDAC and a blueprint for developing PINK1/Parkin or BNIP3/NIX-targeted inhibitors.

#### Blocking autophagosome and autolysosome formation

Once damaged mitochondria are recognized and tagged, a double-membrane structure known as the autophagosome, mediated by LC3 and other autophagy-related proteins, encapsulates them. Subsequently, the autophagosome matures through fusion with a lysosome to form an autolysosome, which then degrades the engulfed mitochondria. PI3K inhibitors, such as 3-methyladenine (3-MA), wortmannin, and LY294002, target autophagy by inhibiting Vps34, a component of class III PI3K, thereby blocking the formation of autophagosomes [96]. However, these inhibitors are not used clinically because they have a broad range of activity, high toxicity, and potential off-target effects, which complicate their therapeutic application. Currently, chloroquine (CQ) and hydroxychloroquine (HCQ) are the most established autophagy inhibitors utilized in clinical settings. They function by inhibiting the formation of autolysosomes and are favored due to their well-defined safety profiles and established dosing regimens [97, 98].

Both CQ and HCQ have been shown to enhance the efficacy of many anticancer regimens by disrupting autolysosome formation and impairing autophagy in preclinical animal models. HCQ, a derivative of CQ, is more commonly used today due to its increased water solubility, higher safety profile, and better tolerability. Phase I/II trials indicate that pre-operative autophagy inhibition with HCQ in combination with gemcitabine is safe and well-tolerated [99]. Subsequent studies, including two randomized trials, have shown that adding HCQ to the gemcitabine/nab-paclitaxel treatment regimen can improve response rates [100, 101]. However, this combination did not appear to improve survival for metastatic or advanced PDAC patients [100]. Similarly, the other clinical trial comparing the effects of HCQ with gemcitabine/nab-paclitaxel in a neoadjuvant setting also failed to demonstrate improvements in overall survival and relapse-free survival [101]. While these inconsistent results may be partly influenced by bias associated with relatively small sample sizes, a more critical factor is the substantial heterogeneity of PDAC, as baseline autophagy activity likely varies across tumors. Notably, patients who exhibited significant increases in the autophagy marker LC3-II in PBMCs showed better survival outcomes, suggesting that stronger autophagy inhibition may provide greater benefit [99, 102]. This emphasizes the importance of patient selection in HCQ-based combination strategies and highlights the need for integrated analyses to identify predictive biomarkers that can translate treatment responses into durable survival advantages. To better determine how HCQ can be optimally combined with current therapeutic regimens to maximize benefit, additional clinical trials are ongoing (Table 1). Furthermore, to enhance the impact of HCQ, novel approaches like incorporating carbon monoxide (CO) to increase the effectiveness of autophagy inhibitors and developing new polymer forms of HCQ (PCQ) have been explored [103, 104].

**Table 1:** Ongoing clinical trials of Hydroxychloroquine combined treatments in PDAC.

ClinicalTrials ID	Mitophagy modulators	Combination treatments	Enrollment	Phase	Study start	Status
NCT03825289	Hydroxychloroquine	Trametinib	25	I	2019-01-18	Recruiting
NCT04132505	Hydroxychloroquine	Binimetinib	39	I	2019-10-22	Active, not recruiting
NCT04524702	Hydroxychloroquine	Paricalcitol with Gemcitabine and Nab-Paclitaxel	12	II	2020-09-14	Active, not recruiting
NCT05518110	Hydroxychloroquine	Trametinib	22	II	2023-05-31	Recruiting
NCT04911816	Hydroxychloroquine	mFOLFIRINOX	40	I/II	2021-07-16	Recruiting
NCT04787991	Hydroxychloroquine	Ipilimumab with Gemcitabine and Nab Paclitaxel	45	I	2021-08-09	Recruiting
NCT05083780	Hydroxychloroquine	Chlorphenesin Carbamate and mFOLFIRINOX	40	I	2021-11-30	Active, not recruiting
NCT05733000	Hydroxychloroquine	CPI-613 with 5-fluorouracil or Gemcitabine	94	II	2023-03-08	Recruiting

In addition to chemotherapy based on gemcitabine and/or nab-paclitaxel, targeted therapy that addresses KRAS mutation, particularly KRAS-G12D, represents another promising strategy [3]. The first-in-class, potent, and selective KRAS-G12D inhibitor, MRTX1133, has demonstrated significant tumor regression in both KRAS-G12D mutant cell line-derived and patient-derived xenograft models, which has led to the initiation of a Phase I/II clinical trial (NCT05737706) [105]. Recently, acquired resistance against this novel KRAS-G12D-targeted therapy through enhanced autophagy has been reported in PDAC [106]. Specifically, autophagy induced by MTOR signaling suppression promotes resistance to MRTX1133 by boosting glutathione synthesis [106]. The combination of CQ overcomes MRTX1133 resistance and completely inhibits in vivo tumor growth.

### Therapeutic strategies: promoting mitophagy

Although numerous studies have demonstrated that mitophagy promotes chemoresistance in cancers, there is also evidence suggesting that promoting mitophagy could, under specific circumstances, enhance chemotherapy-induced cell death. Mitophagy is typically induced by compromising mitochondrial integrity, often through the use of OXPHOS inhibitors. Mitochondrial complex I (CI), the first component in the electron transport chain (ETC), has become a notable target for cancer therapy [107]. Among the most common CI inhibitors known for their anti-cancer activity are the biguanides, particularly metformin. Metformin is commonly used as a diabetes medication that helps make the body more sensitive to insulin. Additionally, it can reversibly inhibit mitochondrial CI and trigger mitophagy, positioning it as a focal point of study for its potential as a tumor suppressor [108, 109]. Metformin-induced mitophagy is mainly mediated by the PINK1/Parkin pathway [110]. In PDAC, metformin combined with gemcitabine has been shown to reduce sphere formation in vitro and enhance apoptosis in cancer cells [111, 112]. However, clinical outcomes have been mixed. While some retrospective studies suggest that metformin may confer a survival benefit for diabetic patients with solid tumors, including PDAC, randomized controlled trials have not found significant benefits for PDAC patients when metformin is combined with gemcitabine [109, 113]. Additionally, a recent observational study reports that metformin may enhance antitumor immunity and improve prognosis in patients with upfront resected PDAC, but not in patients with gemcitabine-based neoadjuvant chemoradiotherapy [114]. Furthermore, another biguanide, phenformin, has demonstrated a synergistic effect with gemcitabine in PDAC cells with high OXPHOS, but not in those with low OXPHOS, through CI inhibition [115]. This may partly explain the variable anti-cancer effects observed with metformin, further underscoring the importance of patient selection in PDAC management due to its significant heterogeneity.

Recently, the novel CI inhibitor IACS-010759 has demonstrated a promising inhibitory effect on cancer cells with high OXPHOS dependency, including PDAC [116]. It has been approved by the FDA for several Phase I clinical trials in advanced cancers [107]. However, it seems to have a narrow therapeutic index with emergent dose-limiting toxicities, which leads to the discontinuation of the trials [117]. Additionally, miriplatin-loaded liposomes (LMPt) have been identified as novel inducers of mitophagy through inhibition of mtDNA replication and subsequent activation of the PINK1/Parkin pathway [118]. The authors suggest that LMPt is a less toxic and more effective chemotherapeutic agent, demonstrating significant anti-cancer activity in PDAC. Furthermore, WJ460, a myoferlin inhibitor, acts as a potent inducer of mitophagy by compromising mitochondrial network integrity [119]. WJ460-induced mitophagy leads to the accumulation of labile iron and lipid peroxidation, which sensitize PDAC cells to ferroptosis.

## Biomarkers and patient selection

Mitophagy plays a context-dependent dual role in PDAC pathogenesis and therapy resistance. Consequently, while combining mitophagy inhibition with chemotherapy shows promise, the results are inconsistent, underscoring the necessity for biomarker-driven patient stratification.

### Single-gene biomarkers

Current efforts to identify reliable biomarkers of mitophagy activity that are capable of predicting prognosis and therapeutic response in PDAC remain a critical unmet challenge. While expression levels of canonical mitophagy markers such as PINK1 [72, 120], BNIP3 [121, 122], and LC3 [123, 124] in tumor tissues are frequently correlated with clinical outcomes, their utility is limited by conflicting prognostic associations. In most malignancies, elevated expression of these markers correlates with poor survival [72, 122]. Paradoxically, however, reduced expression of specific mitophagy regulators, including PRKN [58] and BNIP3 [60], has been linked to worse prognosis in PDAC, underscoring the limitations of monogenic biomarkers in reflecting the functional state of mitophagy.

### Multi-gene prognostic signatures

Recent advances in transcriptomic profiling have enabled the development of multi-gene prognostic models. For instance, a 3-gene signature (PRKN, SRC, VDAC1) stratifies PDAC patients into risk groups, with high-risk scores (defined by elevated SRC/VDAC1 and reduced PRKN expression) predicting poorer survival but enhanced sensitivity to paclitaxel and erlotinib [125]. Complementary work using Reactome-derived mitophagy gene sets classified PDAC into low, intermediate, and high mitophagy subtypes, with the high-mitophagy group exhibiting the worst prognosis and a metabolic shift toward glycolysis [126]. These studies collectively underscore the potential of mitophagy-related gene signatures as prognostic tools in PDAC.

### Metabolic phenotyping

Metabolic heterogeneity is a critical determinant of chemotherapy resistance in PDAC. Metabolomic profiling studies have classified PDAC tumors into several distinct metabolic subtypes, including glycolytic, lipogenic, and quiescent [127–130], which adds a layer of complexity to targeting mitophagy. This metabolic heterogeneity is likely a key determinant of response to therapies that target mitochondria and mitophagy, as evidenced by phenformin’s synergy with gemcitabine exclusively in high-OXPHOS cells [115]. Therefore, metabolic phenotyping, through techniques like transcriptomics, metabolomics, or functional imaging (e.g., FDG-PET), could be a crucial strategy for identifying patients most likely to benefit from mitophagy-directed therapies.

## Conclusions and future perspectives

Mitophagy, the selective degradation of mitochondria, plays a paradoxical role in PDAC. In the premalignant stage, PINK1 or PRKN deletion accelerates tumor progression, while NIX depletion inhibits it. In advanced PDAC, PINK1-mediated mitophagy promotes chemoresistance, while BNIP3-mediated mitophagy is associated with chemosensitivity. These observations underscore the profoundly context-dependent and pathway-specific nature of mitophagy in PDAC, highlighting its potential as a therapeutic target for combination therapy.

However, several challenges must be overcome before clinical translation. First, a deeper understanding of the pathway-specific roles and complex crosstalk between PINK1/Parkin and receptor-mediated mitophagy across diverse PDAC contexts is essential. This will require sophisticated in vivo models enabling conditional and pathway-specific genetic manipulation. Second, the absence of direct, potent, and selective small-molecule modulators has hindered both mechanistic studies and therapeutic development. Current inhibitors like CQ and HCQ are known to affect autophagy; their specific impact on mitophagy remains unclear. The development of targeted compounds, such as those designed to modulate PINK1 phosphorylation and substrate binding, disrupt the BNIP3/NIX homodimer interface, or act as specific LIR motif competitors, remains highly challenging. Small-molecule drugs targeting mitochondrial membrane proteins typically require more sophisticated delivery systems than clinically used tyrosine kinase inhibitors or monoclonal antibodies. In addition, the physiological functions of mitochondria-rich normal cells, such as hepatocytes, cardiomyocytes, and neurons, may be adversely affected. Emerging AI-based protein structure prediction technologies may help overcome these obstacles, offering new avenues for the development of mitophagy-targeted therapeutics. Third, the significant heterogeneity of PDAC dictates that the ultimate challenge is successful patient stratification. Although metabolic subtypes defined by transcriptomic sequencing [127] or plasma metabolomic signatures [131] have been associated with clinical prognosis in PDAC, a mitophagy-based metabolic classification system is not yet mature. Encouragingly, baseline autophagy levels measured from peripheral blood have been reported to correlate with responses to hydroxychloroquine-based combination therapies, suggesting a potentially simple and effective strategy for patient stratification and personalized treatment [99, 102]. Nevertheless, further validation in larger clinical cohorts is required before such an approach can be broadly implemented. In addition to liquid biopsy, tumor tissue biopsy-guided precision medicine in the multi-omics era also enables the development of novel metabolism-based therapeutic strategies, offering new opportunities to overcome treatment resistance in PDAC [132]. Overall, targeting mitophagy in PDAC holds promise, but clinical translation will require pathway-specific insights, selective modulators, advanced delivery strategies, and effective patient stratification.

## Data Availability

No new datasets were analyzed or generated in this review article.
